# Tirzepatide mitigates Stroke-Induced Blood-Brain barrier disruption by modulating Claudin-1 and C/EBP-α pathways

**DOI:** 10.1186/s10020-025-01312-4

**Published:** 2025-07-23

**Authors:** Duozi Wang, Jianhong Wang, Binghu Li, Shu Yang, Fuqiang Guo, Bo Zheng, Jian Wang

**Affiliations:** 1 Department of Neurology, the Affiliated Hospital of University of Electronic Science and Technology, Sichuan Provincial People’s Hospital, Chengdu, 610072 Sichuan China; 2 Department of Neurology, Ya’an Peoples Hospital, No.9 Ankang Road, Ya’an, 625000 Sichuan China

**Keywords:** Stroke, Tirzepatide, Blood-brain barrier (BBB), Claudin-1, C/EBP-α

## Abstract

**Background:**

Stroke is a major cause of disability and mortality worldwide, with ischemic stroke (IS) being the most common form. The blood-brain barrier (BBB) plays a critical role in protecting the brain, and its dysfunction after stroke exacerbates neuronal damage. Therefore, restoring BBB integrity is a promising therapeutic strategy. Tirzepatide (TZP), a dual GLP-1 and GIP receptor agonist, has demonstrated neuroprotective effects, but its role in BBB restoration post-stroke remains unclear.

**Objective:**

This study aims to evaluate the potential of TZP in preventing BBB dysfunction and restoring its integrity in ischemic stroke models.

**Methods:**

Using a middle cerebral artery occlusion (MCAO) mouse model of ischemic stroke, we assessed the effects of TZP on neurological deficits, BBB permeability, and the expression of tight junction (TJ) proteins, particularly Claudin-1. In vitro, human brain microvascular endothelial cells (HBMVECs) were subjected to oxygen-glucose deprivation/reperfusion (OGD/R) to simulate ischemic conditions. The involvement of C/EBP-α, a key transcription factor regulating TJ proteins, was also investigated.

**Results:**

TZP treatment significantly improved neurological scores and reduced BBB permeability in MCAO mice. It also restored Claudin-1 expression, which was downregulated in stroke conditions. In vitro, TZP reduced endothelial permeability and enhanced Claudin-1 expression in OGD/R-treated HBMVECs. Silencing C/EBP-α abolished the protective effects of TZP on both BBB integrity and Claudin-1 expression, indicating that C/EBP-α signaling is crucial for TZP’s action.

**Conclusion:**

TZP ameliorates BBB dysfunction and protects against ischemic stroke by activating C/EBP-α signaling and restoring Claudin-1-mediated tight junction integrity. These findings suggest that TZP holds promise as a therapeutic agent for stroke, offering a novel strategy for maintaining BBB function and reducing neuronal damage. Further studies are needed to explore the detailed mechanisms underlying TZP’s neuroprotective effects and its clinical potential in stroke therapy.

**Supplementary Information:**

The online version contains supplementary material available at 10.1186/s10020-025-01312-4.

## Introduction

Stroke, also referred to as a cerebrovascular accident, represents a significant neurological disorder characterized by high rates of disability and mortality. It stands as one of the leading causes of death globally in recent years. With the aging population on the rise, coupled with increased exposure to risk factors such as hypertension, hyperglycemia, hyperlipidemia, environmental particulate pollution, and elevated body mass index, the incidence of stroke has been escalating annually (Hilkens et al. 2024; Janas et al. 2023). According to the most recent epidemiological surveys, approximately 2.4 million new stroke cases are diagnosed in China each year, with the majority of survivors experiencing varying degrees of neurological dysfunction. This imposes a substantial burden on patients, their families, and society at large (Tu et al. 2023; Tu and Wang 2023). Ischemic stroke (IS), being the most prevalent form of stroke, is currently primarily treated through intravenous thrombolysis, and mechanical thrombectomy, alongside the use of neurotrophic drugs, circulatory improvement agents, and antiplatelet medications. However, due to the limited therapeutic window and severe associated complications, a majority of patients do not qualify for these interventions (Rabinstein 2020; Herpich and Rincon 2020). Consequently, identifying novel therapeutic strategies for IS remains a focal point in clinical research.

The blood-brain barrier (BBB) constitutes a protective shield formed by vascular endothelial cells (ECs) and neuroglial cells, separating blood from brain parenchyma or cerebrospinal fluid. Its primary function is to obstruct the entry of potentially harmful substances into the brain, thereby safeguarding neural cells (Kadry et al. 2020). Following an IS event, a cascade of pathological alterations ensues within the BBB. Hypoxia and ischemia trigger extensive neuronal cell apoptosis, while polarized resting microglia secrete chemokines, attracting a multitude of immune cells from the circulation into the brain tissue. Throughout this process, astrocytes, microglia, and pericytes, among others undergo phenotypic shifts. Accumulated immune cells release a plethora of pro-inflammatory cytokines (IL-6, TNF-α, IL-1β), reactive oxygen species (ROS), reactive nitrogen species (RNS), matrix metalloproteinases (MMPs), and nitric oxide synthase (NOS), which collectively promote further neural cell death, disrupt ionic homeostasis, and exacerbate cerebral edema (Turner and Sharp 2016; Lv et al. 2018; Xu et al. 2021). The integrity of capillary ECs weakens, leading to a gradual detachment of astrocytic end-feet, heightening BBB permeability. This permits the accumulation of macromolecules within brain cells, exacerbating the disruption of the cerebral microenvironment and amplifying injury (Liu et al. 2018; Parvez et al. 2022; Song et al. 2021). Thus, reinforcing BBB repair mechanisms emerges as a pivotal strategy in IS management.

Tirzepatide (TZP) is an innovative drug designed to regulate blood glucose levels via dual agonism of Glucose-Dependent Insulinotropic Polypeptide (GIP) and Glucagon-Like Peptide-1 (GLP-1) receptors (Forzano et al. 2022). Unlike GLP-1 analogs such as liraglutide and semaglutide, which show limited BBB penetration (Kp, brain ~ 0.02–0.05) (Rhea et al. 2024; Lee et al. 2023; Hunter and Hölscher 2012; Secher et al. 2014), TZP’s dual agonism and fatty acid modification may enhance its BBB permeability. This enhancement offers a novel approach for IS therapy and is the focus of this study, which addresses the gap in understanding TZP’s effects on BBB restoration post-stroke compared to single GLP-1 agonists. Its structural basis lies in the sequence of endogenous GIP, and its pharmacological impact on GLP-1 receptors mirrors that of native GIP but with enhanced potency. A 72-week clinical trial demonstrated that weekly TZP administration can induce substantial and sustained weight loss (Venniyoor 2022). Moreover, TZP exhibits neuroprotective potential by modulating cerebral glucose metabolism (Yang et al. 2024). As a synthetic linear peptide comprising 39 amino acids, TZP’s modification with fatty acids bolsters its stability and therapeutic efficacy (Forzano et al. 2022). Recent studies have unveiled TZP’s neuroprotective properties in APP/PS1 mouse models, where it mitigates neuroinflammation and neuronal apoptosis induced by β-amyloid through enhancements in brain glucose metabolism and mitochondrial function (Yang et al. 2024). This investigation delves into the prospects of TZP as a treatment for IS by examining its influence on BBB restoration and the underlying regulatory mechanisms.

## Methods

### Animals

The ICR mice, weighing between 23 and 25 g, were sourced from the Laboratory Animal Center at Zhejiang Chinese Medical University. The CAG-CreERT^2^ mouse line was acquired from Jackson Laboratory. Additionally, C/EBPα-floxed mice were obtained following the procedures outlined in previous studies (Li et al. 2001; Lee et al. 1997). To generate C/EBPα-deficient (C/EBPα-/-) mice, we crossed the C/EBPα-floxed mice with the CAG-CreERT^2^ strain. The study protocol was approved by the Ethics Committee of Ya’an Peoples Hospital. All efforts were made to minimize animal suffering and to reduce the number of animals used in the experiments.

### Middle cerebral artery occlusion (MCAO) modeling

Mice were anesthetized with 4% chloral hydrate (10 mL/kg) and then secured in a supine position. A midline incision was made in the neck, the right submandibular gland was retracted outward, and the common carotid artery’s pulsation was visualized within the triangle formed by the left sternohyoid muscle, sternothyroid muscle, and digastric muscle. The typical carotid artery, external carotid artery, and internal carotid artery were carefully isolated and exposed. A tie was placed proximally on the typical carotid artery near the heart, while the external carotid artery was ligated at its distal end and transected further downstream. A segment of the internal carotid artery was freed along its length. A ligature was left intact near the heart end of the external carotid artery. Both the external carotid artery and the distal end of the common carotid artery were then ligated, and a small puncture was made at the distal end of the external carotid artery using a No. 4.5 needle. A prepared line embolus (a 6 − 0 nylon monofilament line, approximately 20 mm long, with a uniform silicone coating of 3 mm at the front end, a final diameter of about 0.2 mm, and a blunt tip) was inserted. The line was navigated through the bifurcation of the common carotid artery into the internal carotid artery. The angle of insertion was adjusted to avoid mistakenly entering the palatine artery. The line was advanced until slight resistance was felt, reaching a depth of 8 to 9 mm beyond the bifurcation, indicating that approximately 11 mm of the line was inserted, with the head of the line just entering the anterior cerebral artery by about 1 mm. After the line was properly positioned, the previously placed ligature on the external carotid artery was tightened to secure the line and stop bleeding. The neck wound was then routinely sutured. One hour after MCAO induction, the mice were re-anesthetized, the external carotid artery was re-exposed, the fixing ligature was loosened, the line embolus was removed, and the superficial carotid vessel was electrocoagulated, while the ties on the main carotid vessel were released. Good pulsation of both the common carotid artery and the internal carotid artery indicated successful reperfusion. Throughout the surgical procedure, the animal’s anal temperature was maintained at 37 °C. Post-surgery, the animals were housed in clean cages with unrestricted access to food and water. In the Sham surgery group, the line embolus was immediately removed upon reaching the corresponding depth, and the rest of the procedures were identical to those for the MCAO mice.

### MCAO and validation

Male ICR mice (23–25 g) underwent MCAO under 2% isoflurane, occluding the right middle cerebral artery for 1 h with a 6 − 0 nylon suture (Doccol, 602234PK10), followed by reperfusion. MCAO was validated by cortical blanching during occlusion and neurological deficits (Bederson score ≥ 2, assessed 24 h post-MCAO). Sham mice underwent vessel exposure without occlusion. TZP (10 nmol/kg, i.p., Eli Lilly) or saline was administered weekly for 8 weeks, starting 24 h post-MCAO.

### TTC staining

Twenty-four hours following MCAO, cerebral tissues were coronally sliced into 2 mm thick sections. These sections were incubated in a 2% 2,3,5-triphenyltetrazolium chloride (TTC; Sigma-Aldrich, USA) solution at 37 °C for 20 min to visualize ischemic regions. Infarct volume analysis was performed using ImageJ (NIH, USA), with results normalized as a percentage of the contralateral hemisphere’s area to account for edema and tissue shrinkage.

### Animal grouping

To evaluate the therapeutic function of TZP against IS, four groups were divided: Sham, TZP, MCAO, and MCAO + TZP groups. In the Sham and MCAO groups, normal saline was intraperitoneally administered to sham-operated and MCAO-operated wide-type (WT) mice once a week for 8 weeks, respectively. In the TZP and MCAO + TZP groups, sham-operated and MCAO-operated WT mice were intraperitoneally administered with 10 nmol/kg TZP once a week for 8 weeks, respectively. To confirm the involvement of C/EBPα in the function of TZP, four groups were divided: Sham, MCAO, MCAO + TZP, and MCAO + C/EBP-α-/- + TZP. Administrations in the Sham, MCAO, and MCAO + TZP groups were described above. MCAO-operated C/EBP-α-/- mice in the MCAO + C/EBP-α-/- + TZP group received an intraperitoneal injection of 10 nmol/kg TZP every week for eight weeks.

### Neurological scoring

After treatments, neurological scores were assigned using Longa’s 5-point scoring system to assess the severity of behavioral impairments in animals, which indirectly reflected the extent of brain neurological damage. The scoring criteria were as follows: 0 points, no neurological deficits; 1 point, inability to fully extend the contralateral forepaw; 2 points, circling towards the contralateral side; 3 points, falling towards the contralateral side; 4 points, inability to walk spontaneously or loss of consciousness.

### BBB permeability assay

^14^C-sucrose (1 µCi, i.v.) was administered, and brain uptake was measured after 10 min. The influx rate (Ki, µL/g/min) was calculated as [^14^C-sucrose]brain (dpm/g)/[^14^C-sucrose]plasma (dpm/mL) × time (min).

### Brain-to-Plasma ratio measurement

TZP concentrations were measured in plasma and brain from Sham, TZP, MCAO, and MCAO + TZP groups (*n* = 6 per group) after 8 weeks of treatment (TZP: 10 nmol/kg, saline: 0.1 mL, i.p., weekly). At 24 h post-final dose, mice were anesthetized (xylazine 8 mg/kg, ketamine 140 mg/kg, i.p.). Plasma was collected via cardiac puncture (0.5 mL, heparinized tubes) and centrifuged (3,000 × g, 10 min, 4 °C). The right cerebral hemisphere was harvested, rinsed in cold saline, weighed, and homogenized in 1 mL PBS with 1% protease inhibitor. Homogenates were centrifuged (10,000 × g, 15 min, 4 °C). TZP was quantified by LC-MS/MS using a Shimadzu LC-20AD system and AB Sciex QTRAP 5500. Samples were diluted 1:10 in acetonitrile with internal standard (semaglutide-d6, 10 ng/mL), separated on a C18 column (2.1 × 100 mm, 1.8 μm), and detected via multiple reaction monitoring (MRM). Calibration curves (0.1–100 ng/mL, r² > 0.99) ensured linearity. Kp, brain was calculated as [TZP]brain (ng/mL, converted from ng/g)/[TZP]plasma (ng/mL). Sham and MCAO groups (no TZP) served as controls. Liraglutide’s Kp, brain was cited from the literature (Rhea et al. 2024).

### Immunohistochemical analysis

Mouse brain cortex samples were traditionally fixed in a 10% formaldehyde solution, embedded in paraffin, sectioned, and then dehydrated. Endogenous peroxidase activity was quenched by incubating the sections with a 3% hydrogen peroxide solution for 30 min. Antigen retrieval was carried out using ethylenediaminetetraacetic acid (EDTA) under pressure. After sealing for 30 min, slides were incubated overnight at 4 °C with primary antibodies specific for Claudin-1 (Abcam, USA), diluted at a ratio of 1:400. This incubation period was extended to ensure thorough binding. Subsequently, the slides were washed and incubated with secondary antibodies (1:400, Abcam, USA) for 15 min. Finally, the slides were developed using a DAB chromogenic substrate system, counterstained with hematoxylin, differentiated with an ethanol solution containing hydrochloric acid, subjected to a bluing step with running tap water, rinsed with deionized water, dehydrated, and covered with a protective cover slip. Visual documentation was obtained using a microscope (Zeiss, Germany).

### RT-PCR assay

1 mL of RNAiso Plus solution was added to the pulverized rodent cerebral cortex samples or cellular material. Following complete homogenization, the liquid fraction was extracted after high-speed centrifugation. The overall RNA pellet was then washed with 75% ethanol, and the RNA was subsequently harvested for reverse transcription. These specific procedures were carried out in accordance with the instructions provided in the PrimeScript RT reagent Kit (TaKaRa, Japan). Real-time PCR detection was performed following the experimental protocols of the Applied Biosystems 7300 instrument. Gene expression levels were determined based on cycle threshold counts.

### Western blot analysis

Claudin-1 and C/EBP-α were analyzed in brain homogenates (right hemisphere: Sham, MCAO, MCAO + TZP) and HBMVECs (Control, OGD/R, OGD/R + TZP). A lysis solution, composed of 1% protease inhibitor in RIPA buffer (Sigma-Aldrich, R0278), was introduced to samples, which were homogenized over ice for 30 min. The homogenate was centrifuged at 13,500 rpm for 10 min at 4 °C. Protein was quantified using the BCA assay kit (Sigma-Aldrich, BCA1), and 50 µg protein/lane was separated on 10% SDS-PAGE and transferred to PVDF in electrophoresis buffer at 200 mA for 2 h. The membrane was blocked with 5% skim milk in TBST for 1 h. Primary antibodies were incubated overnight at 4 °C: Claudin-1 (1:1000, Abcam, ab15098), C/EBP-α (1:1000, Santa Cruz Biotechnology, sc-166258), and GAPDH (1:2000, Sigma-Aldrich, A5441). HRP-conjugated secondary antibodies (1:5000, Santa Cruz, sc-2357) were incubated for 1 h. Bands were detected via ECL (Bio-Rad, 1705061), quantified with ImageJ, and normalized to GAPDH.

### Cells and treatments

HBMVECs were sourced from ATCC (USA). These cells were cultivated in a medium specifically designed for endothelial cultures, enriched with 10% FBS, and maintained in an incubator set at a temperature of 37 °C with 5% CO_2_. For OGD/R modeling, the culture medium was replaced with a serum-free, glucose-free medium, and then it was placed in a hypoxic environment (1% O_2_, 5% CO_2_, 94% N_2_) at a 37 °C incubator for 10 h. After 10 h, the normal medium containing serum was added, and the normal oxygen supply was restored. The cells were then kept in a 37 °C constant temperature incubator for 24 h of reoxygenation. To silence C/EBP-α, HBMVECs were transduced with adenovirus harboring a short hairpin RNA (shRNA) specifically designed to knock down C/EBP-α (Ad-viral sh-C/EBP-α) for 48 h.

### TEER value determination

Approximately 100,000 cells were nurtured in the upper section of the Transwell device. Once the cells had bonded to the container surfaces, TEER values across each group were determined utilizing an impedance meter (Millipore, United States) after 24 h.

### FITC-dextran permeation experiments

A serum-free medium was used to dilute FITC-dextran to various concentrations for the establishment of a calibration curve. Subsequently, HBMVECs were cultured in the upper section of the Transwell setup. Thereafter, 100 µL of a 1 mg/mL FITC-conjugated dextran solution was added to the upper section and allowed to rest for 1 h in a shielded environment at 37 °C with 5% CO_2_. Post incubation, 100 µL of fluid was drawn from the lower section, and the luminescence intensity of the fluid in the lower section was quantified using a luminescence photometer (494/520 nm). Regarding the calibration curve and the observed luminescence intensity, the permeation level of FITC-dextran across the Transwell chamber was computed (µg/mL).

### In vitro transwell bbb permeability assay

HBMVECs were cultured in EBM-2 (Lonza, CC-3156; 10% FBS, EGM-2 BulletKit) and seeded (5 × 10⁴ cells/cm²) on collagen I-coated Transwell inserts (0.4 μm, 12 mm, Corning, 3460). After 7 days (TEER > 200 Ω·cm², EVOM2), groups were tested: Control (0.1% DMSO), TZP (100 nM), OGD/R (2 h glucose-free DMEM, 1% O₂, 24 h reperfusion), OGD/R + TZP (100 nM). TZP/vehicle was added apically at reperfusion. FITC-dextran (70 kDa, 1 mg/mL, Sigma-Aldrich, 46945) was added apically; basolateral fluorescence (490/520 nm, BioTek Synergy H1) was measured after 2 h. Papp (cm/s) = (dQ/dt)/(A × C₀), A = 1.12 cm², C₀ = 1000 µg/cm³. Triplicates ensured CV < 10%. TEER > 150 Ω·cm² post-assay confirmed integrity.

### In vivo P-gp inhibition study

ICR mice (male, 23–25 g, *n* = 6/group) underwent 1-h MCAO. TZP (10 nmol/kg, i.p., weekly, 8 weeks) was given post-24 h. Groups: MCAO + TZP (saline, i.p.), MCAO + TZP + Verapamil (20 mg/kg, i.p., 30 min before final TZP, Sigma-Aldrich, USA). At 24 h post-final dose, plasma (0.5 mL, cardiac puncture) and right hemisphere were collected (anesthesia: xylazine 8 mg/kg, ketamine 140 mg/kg). Samples were homogenized (brain: 1 mL PBS + 1% protease inhibitor), diluted 1:10 in acetonitrile (semaglutide-d6 standard, 10 ng/mL). LC-MS/MS (Shimadzu LC-20AD, AB Sciex QTRAP 5500, C18 column) used multiple reaction monitoring (MRM). Calibration: 0.1–100 ng/mL, r² > 0.99. Kp, brain = [TZP]brain (ng/mL, 1 g ≈ 1 mL)/[TZP]plasma. CV < 10%.

### Statistical analysis

GraphPad Prism 8.0 was employed for statistical analysis and plotting. All data were presented as mean ± standard deviation (SD). For multiple group comparisons, ANOVA was employed, followed by Tukey’s post hoc test for pairwise comparisons. In cases involving comparisons between two groups, unpaired t-tests were utilized. A *P* value of < 0.05 was regarded as statistically significant.

## Results

### TZP improved neurological deficits post-stroke

**Fig. 1:**
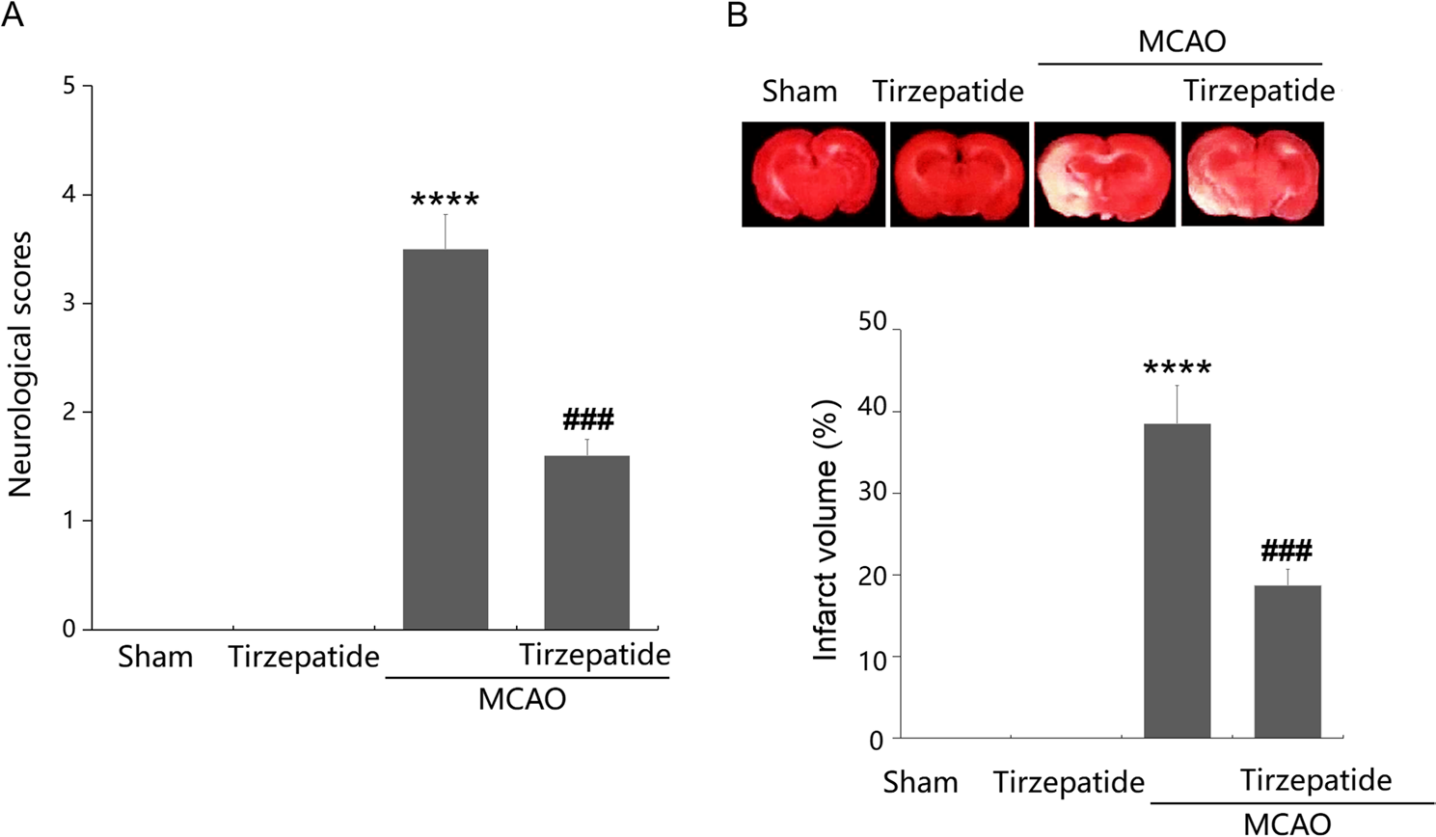
TZP improved neurological deficits post-stroke. Mice were divided into four groups: Sham, TZP, MCAO, and MCAO + TZP groups. **A** Neurological scores were evaluated using Longa’s 5-point scoring system post-MCAO; **B** Representative TTC-stained coronal brain slices are shown. Infarct volume (% of the ipsilateral hemisphere) in Sham, TZP, MCAO, and MCAO + TZP groups (****P < 0.001 vs. sham group; ^###^*P* < 0.005 vs. MCAO group)

To evaluate the therapeutic function of TZP against IS, four groups were divided: Sham, TZP, MCAO, and MCAO + TZP groups. In the Sham and TZP group, neurological scores were maintained at 0, which were markedly elevated to 3.5 in MCAO mice. Following TZP administration, the neurological scores of MCAO mice were sharply reduced to 1.6 (Fig. 1A). TTC staining confirmed MCAO modeling, with MCAO mice showing 38.5 ± 4.2% infarct volume in the ipsilateral hemisphere. TZP reduced infarct volume to 18.7 ± 3.1% in MCAO + TZP mice (Fig. 1B). In summary, TZP significantly improves neurological deficits and mitigates stroke-induced neuronal damage by reducing infarct volume, highlighting its neuroprotective potential in the MCAO model.

### TZP ameliorated BBB dysfunction and restored Claudin-1, ZO-1, and occludin expressions post-stroke

**Fig. 2:**
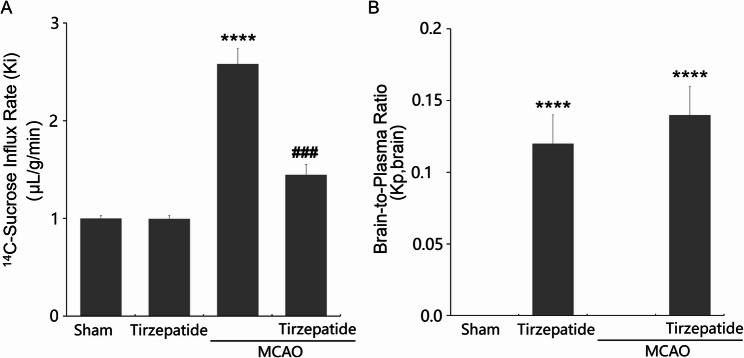
TZP Reduces Stroke-Induced BBB Permeability in MCAO Mice. Mice were divided into 4 groups: Sham, TZP, MCAO, and MCAO + TZP groups. The BBB permeability was assessed using ^14^C-sucrose. **A** ^14^C-sucrose influx rate (Ki, µL/g/min) in the right cerebral hemisphere for Sham, TZP, MCAO, and MCAO + TZP groups (*n* = 6, mean ± SD). ****P < 0.001 vs. Sham; ^###^*P* < 0.005 vs. MCAO (ANOVA with Tukey post hoc). **B** Brain-to-plasma ratio (Kp, brain) for TZP in Sham, TZP, MCAO, and MCAO + TZP groups (*n* = 6, mean ± SD). ****P < 0.001 vs. Sham/MCAO; no significant difference between TZP and MCAO + TZP (*P* > 0.05, ANOVA with Tukey post hoc)

Subsequently, BBB permeability was assessed by ^14^C-sucrose influx rate (Ki) in the right cerebral hemisphere. In Sham mice, Ki was 1.00 ± 0.028 µL/g/min (*n* = 6, mean ± SD). TZP treatment alone (TZP group) yielded a similar Ki of 0.995 ± 0.032 µL/g/min (*P* = 0.89 vs. Sham). In MCAO mice, Ki increased significantly to 2.582 ± 0.156 µL/g/min (*P* < 0.001 vs. Sham), indicating BBB disruption. TZP treatment in MCAO mice (MCAO + TZP) reduced Ki to 1.447 ± 0.103 µL/g/min (*P* < 0.005 vs. MCAO, ANOVA with Tukey post hoc; Fig. 2A). TZP’s brain penetration was assessed by Kp, brain in all groups after 8 weeks. In Sham and MCAO groups (no TZP), TZP was undetectable in plasma and brain. In the TZP group, plasma and brain concentrations were 80.2 ± 10.1 ng/mL and 9.6 ± 1.2 ng/g, yielding a Kp, brain of 0.12 ± 0.02, exceeding the threshold for moderate permeability (Kp, brain > 0.1) (Pardridge 2005). In MCAO + TZP, concentrations were 82.4 ± 12.3 ng/mL and 11.5 ± 1.7 ng/g, yielding a Kp, brain of 0.14 ± 0.02. Kp, brain differed significantly across groups (*P* < 0.001, ANOVA), with TZP and MCAO + TZP higher than Sham/MCAO (*P* < 0.001, Tukey), but TZP vs. MCAO + TZP was not significant (*P* = 0.08). Compared to liraglutide’s reported Kp, brain of 0.03 ± 0.01 (Rhea et al. 2024), TZP’s Kp, brain in both TZP and MCAO + TZP groups was significantly higher (*P* < 0.01), indicating enhanced BBB penetration (Fig. 2B).

**Fig. 3:**
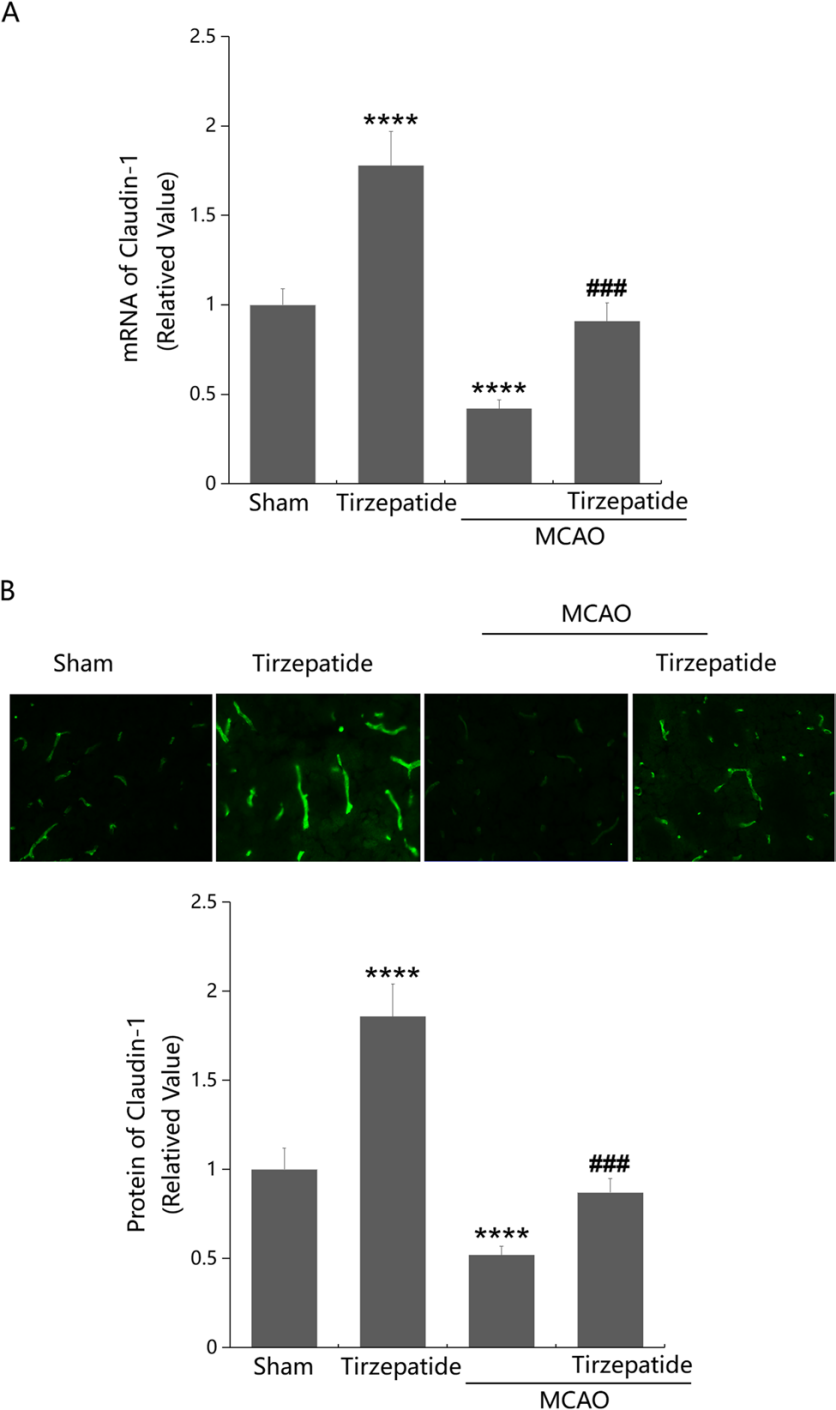
TZP restored the expression of Claudin-1 in the cortex of mice post-stroke. Mice were divided into 4 groups: Sham, TZP, MCAO, MCAO + TZP groups. **A** mRNA of Claudin-1; **B** Protein of Claudin-1 as measured by immunostaining (****P < 0.001 vs. sham group; ^###^*P* < 0.005 vs. MCAO group)

Claudin-1 is a critical TJ protein engaged in BBB function (Zorn-Kruppa et al. 2018). Here, in the cortex tissue, we found that Claudin-1 expressions were remarkably enhanced in the TZP group, and markedly diminished in the MCAO group, which were strikingly enhanced by TZP (Fig. 3A, B).

### TZP activated C/EBP-α signaling in MCAO mice

**Fig. 4:**
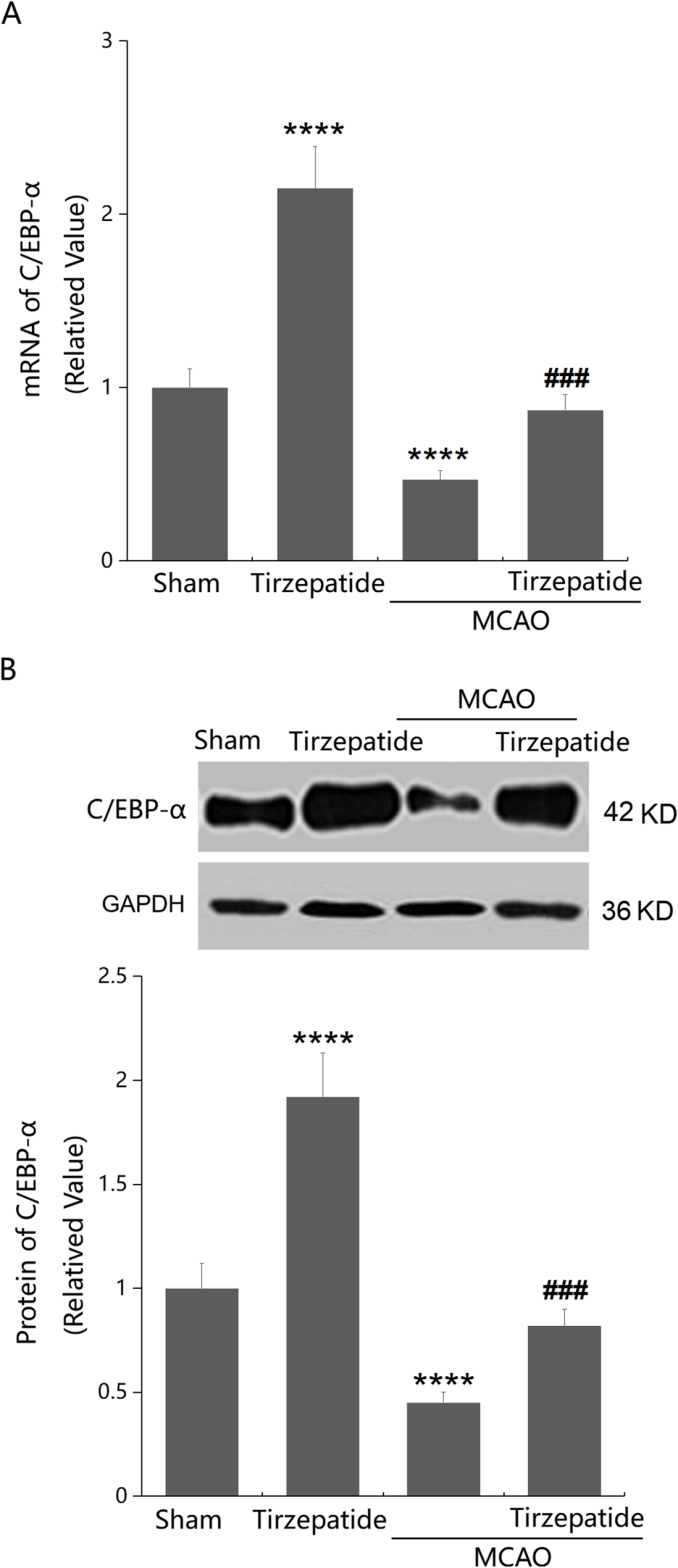
TZP activated C/EBP-α signaling in the cortex of mice post-stroke. Mice were divided into 4 groups: Sham, TZP, MCAO, and MCAO + TZP groups. **A** mRNA of C/EBP-α; **B** Protein of C/EBP-α (****P < 0.001 vs. sham group; ^###^*P* < 0.005 vs. MCAO group)

C/EBP-α signaling is claimed to participate in regulating expressions of TJ proteins (Yang et al. 2024; Li et al. 2001; Lee et al. 1997; Pardridge 2005; Zorn-Kruppa et al. 2018; Kakogiannos et al. 2020). In cortex tissues, C/EBP-α levels were notably elevated in the TZP group, and markedly decreased in the MCAO group, which were largely enhanced by TZP administration (Fig. 4A, B).

### Silencing C/EBP-α abolished the effects of TZP on neurological deficits and BBB permeability post-stroke

**Fig. 5:**
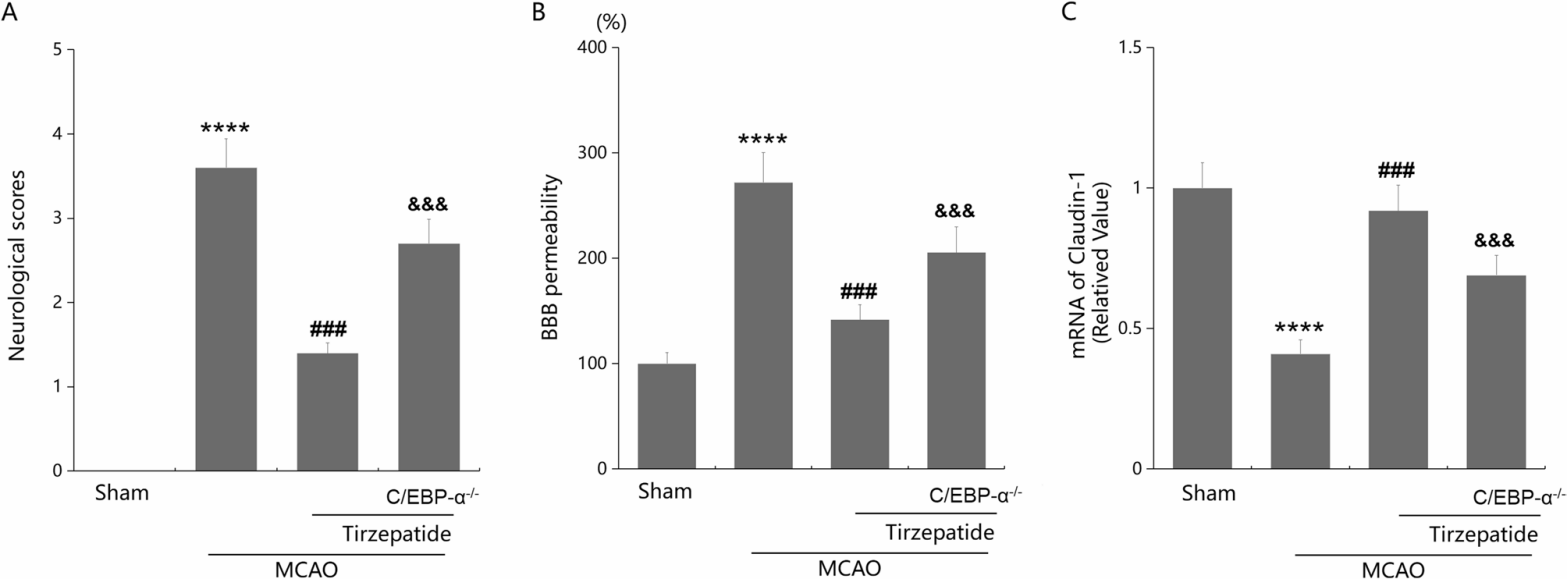
Silencing C/EBP-α abolished the effects of TZP on neurological deficits and BBB permeability post-stroke. Mice were divided into 4 groups: Sham, MCAO, MCAO + TZP, MCAO + C/EBP-α^−/−^ + TZP. **A** Neurological scores were determined 3 days after MCAO; **B** The BBB permeability was assessed using ^14^C-Sucrose; **C** mRNA of Claudin-1 (****P < 0.001 vs. sham group; ^###^*P* < 0.005 vs. MCAO group; ^&&&^*P* < 0.005 vs. MCAO + TZP group)

To confirm the involvement of C/EBP-α in the anti-stroke function of TZP, four groups were divided: Sham, MCAO, MCAO + TZP, and MCAO + C/EBP-α-/- + TZP. Neurological scores in the Sham, MCAO, MCAO + TZP, and MCAO + C/EBP-α-/- + TZP groups were 0, 3.6, 1.4, and 2.7, respectively (Fig. 5A). Furthermore, BBB permeability was sharply increased from 100 to 272.1% in MCAO mice, which was notably restrained to 141.9% by TZP. Compared to TZP-treated MCAO WT mice, the BBB permeability was noticeably enhanced to 205.7% in TZP-treated MCAO C/EBP-α-/- mice (Fig. 5B). Moreover, remarkably downregulated Claudin-1 levels in MCAO mice were sharply enhanced by TZP, which were strikingly repressed by silencing C/EBP-α (Fig. 5C).

#### TZP attenuated OGD/R-induced aggravation of endothelial permeability of HBMVECs and restored Claudin-1 expressions after OGD/R

**Fig. 6:**
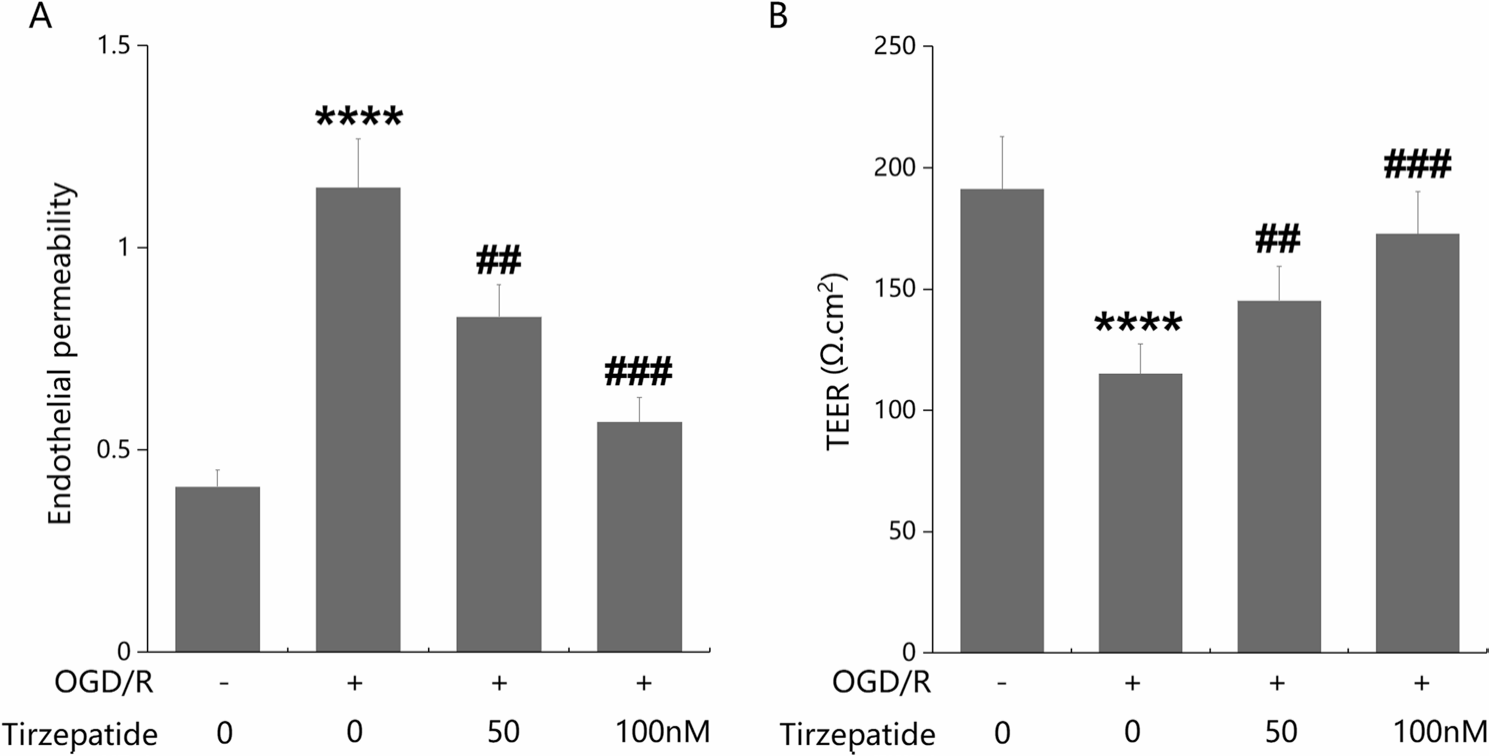
TZP attenuated OGD/R induced aggravation of endothelial permeability of human primary microvascular endothelial cells (HBMVECs) after OGD/R. HBMVECs were challenged by OGD/R with or without TZP (50, 100 nM). **A** Endothelial permeability was assayed by FITC-dextran permeation; **B** The trans-endothelial electrical resistance (TEER) (****P < 0.001 vs. sham group; ^##^, ^###^*P* < 0.01, 0.005 vs. OGD/R group)

**Fig. 7:**
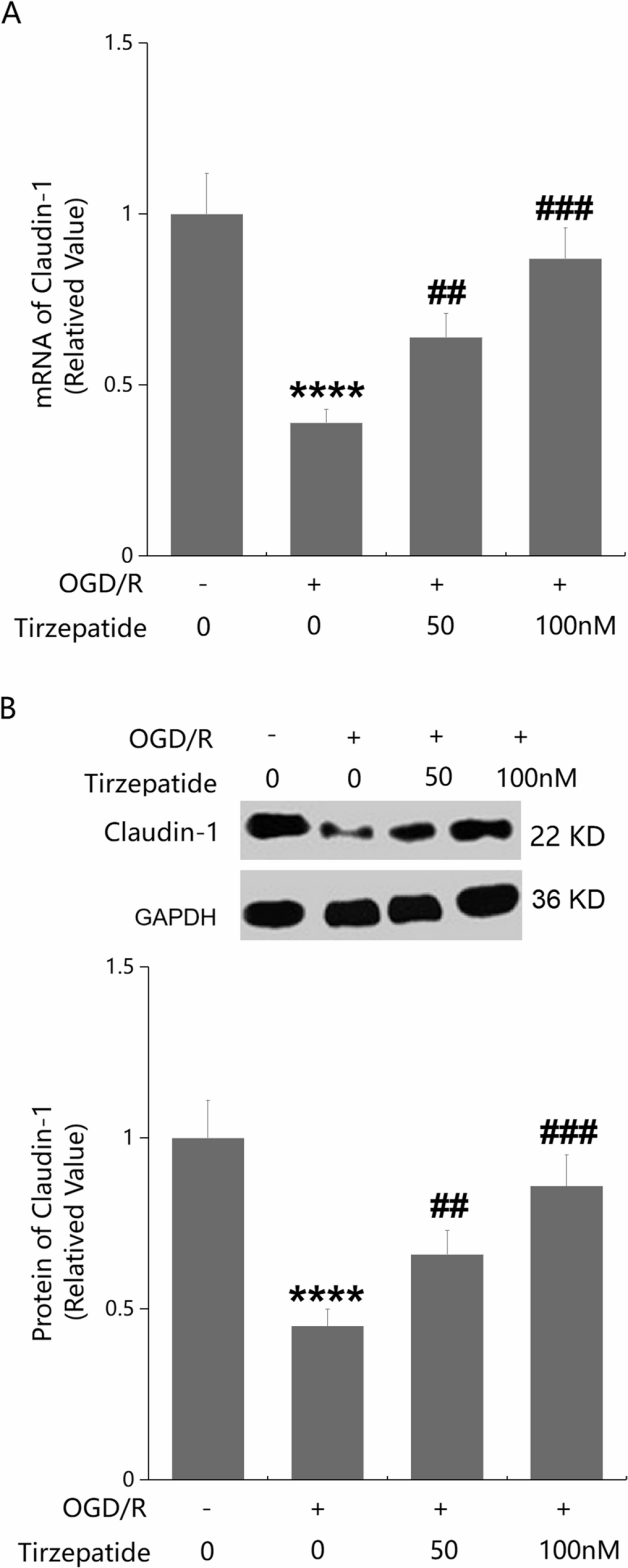
TZP restored Claudin-1 expressions in OGD/R-stimulated HBMVECs. HBMVECs were challenged by OGD/R with or without TZP (50, 100 nM). **A** mRNA of Claudin-1 as measured by real-time PCR; **B** Protein of Claudin-1 as measured by western blot analysis (****P < 0.001 vs. sham group; ^##^, ^###^*P* < 0.01, 0.005 vs. OGD/R group)

HBMVECs were challenged by OGD/R with or without TZP (50, 100 nM) for 24 h. In the endothelial permeability study, FITC concentrations in the lower chamber were strikingly increased from 0.41 to 1.15 mg/mL, which were noticeably diminished to 0.83 and 0.57 mg/mL by 50 and 100 nM TZP respectively (Fig. 6A). Moreover, TEER values in the control, OGD/R, 50 nM TZP, and 100 nM TZP groups were 191.3, 115.3, 145.3, and 172.9 Ω.cm², respectively (Fig. 6B). In HBMVECs, Claudin-1 expressions were strikingly diminished, which were notably enhanced by 50 and 100 nM TZP consistent with in vivo data (Fig. 7A, B).

### The protective effects of TZP in inhibiting endothelial permeability and Claudin-1 expression are mediated through C/EBP-α

**Fig. 8:**
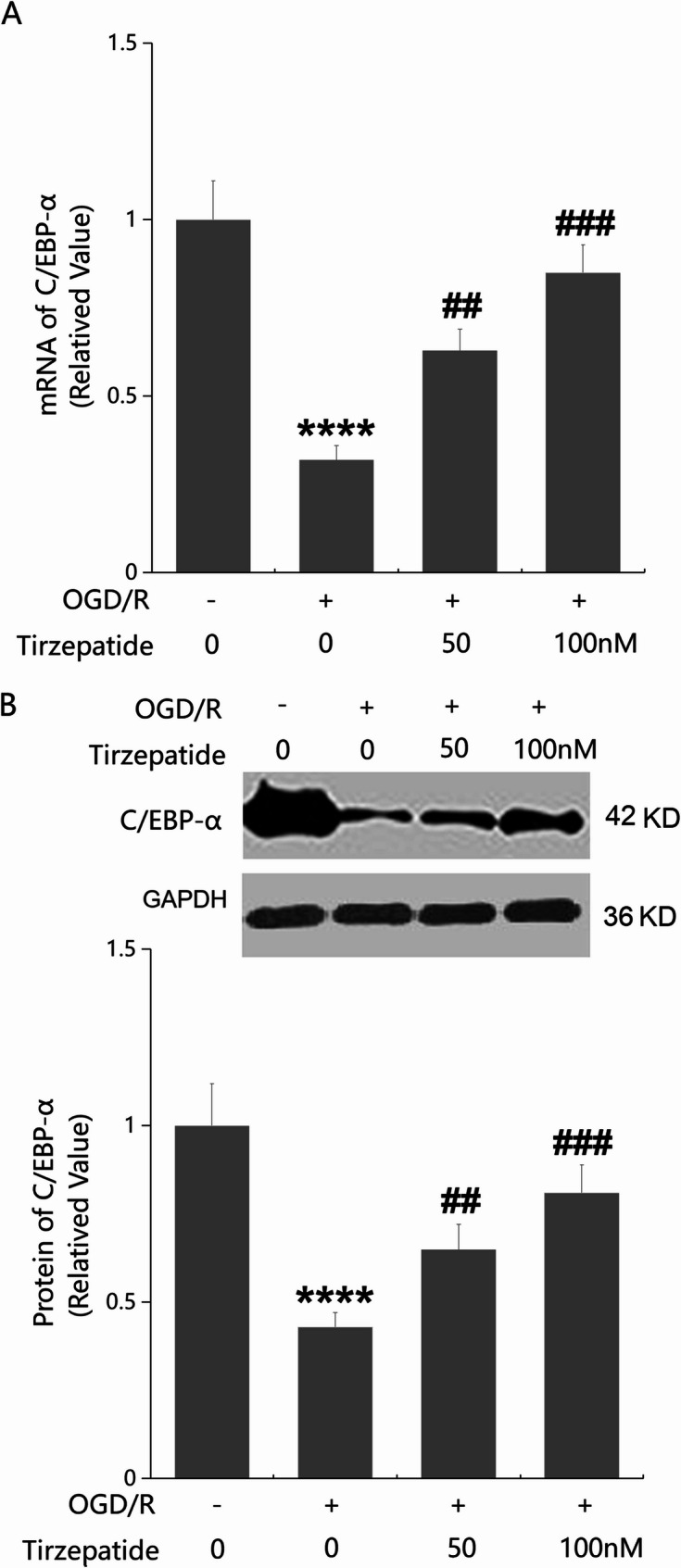
TZP activated C/EBP-α signaling in OGD/R-stimulated HBMVECs. HBMVECs were challenged by OGD/R with or without TZP (50, 100 nM). **A** mRNA of C/EBP-α as measured by real-time PCR; **B** Protein of C/EBP-α as measured by western blot analysis (****P < 0.001 vs. sham group; ^##^, ^###^*P* < 0.01, 0.005 vs. OGD/R group)

**Fig. 9:**
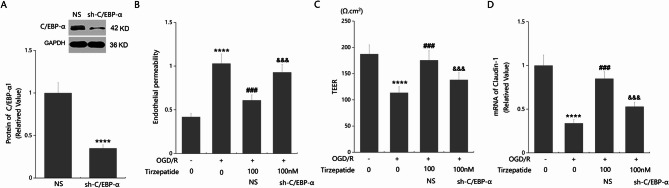
Silencing C/EBP-α abolished the effects of TZP in protecting endothelial permeability. HBMVECs were transduced with Ad-viral sh-C/EBP-α, followed by challenge with OGD/R with or without TZP (100 nM). **A** Successful knockdown of C/EBP-α as measured by western blot analysis; **B** Endothelial permeability was assayed by FITC-dextran permeation; **C** The trans-endothelial electrical resistance (TEER). **D** mRNA of Claudin-1 as measured by real time PCR (****P < 0.001 vs. sham group; ^###^*P* < 0.005 vs. OGD/R group; ^&&&^*P* < 0.005 vs. OGD/R + TZP group)

In HBMVECs, C/EBP-α levels were remarkably repressed, which were largely promoted by 50 and 100 nM TZP consistent to in vivo data (Fig. 8A, B). To confirm the mechanism, HBMVECs were transduced with Ad-viral sh-C/EBP-α, followed by challenge with OGD/R with or without TZP (100 nM). The successful knockdown of C/EBP-α was validated by Western blot analysis (Fig. 9A). FITC concentrations in the lower chamber in the endothelial permeability study in the control, OGD/R, OGD/R + TZP, and OGD/R + sh-C/EBP-α + TZP groups were 0.42, 1.03, 0.61, and 0.93 mg/mL, respectively (Fig. 9B). TEER values in HBMVECs were reduced from 187.4 to 113.5 Ω.cm² by OGD/R which were largely enhanced to 175.6 Ω.cm² by TZP. Following silencing C/EBP-α, TEER values were reversed to 138.2 Ω.cm² (Fig. 9C). In addition, diminished Claudin-1 expressions in OGD/R managed HBMVECs were remarkably elevated by TZP, which were strikingly restrained by silencing C/EBP-α (Fig. 9D).

### In vitro and in vivo validation of TZP’s BBB protection and CNS penetration

**Fig. 10:**
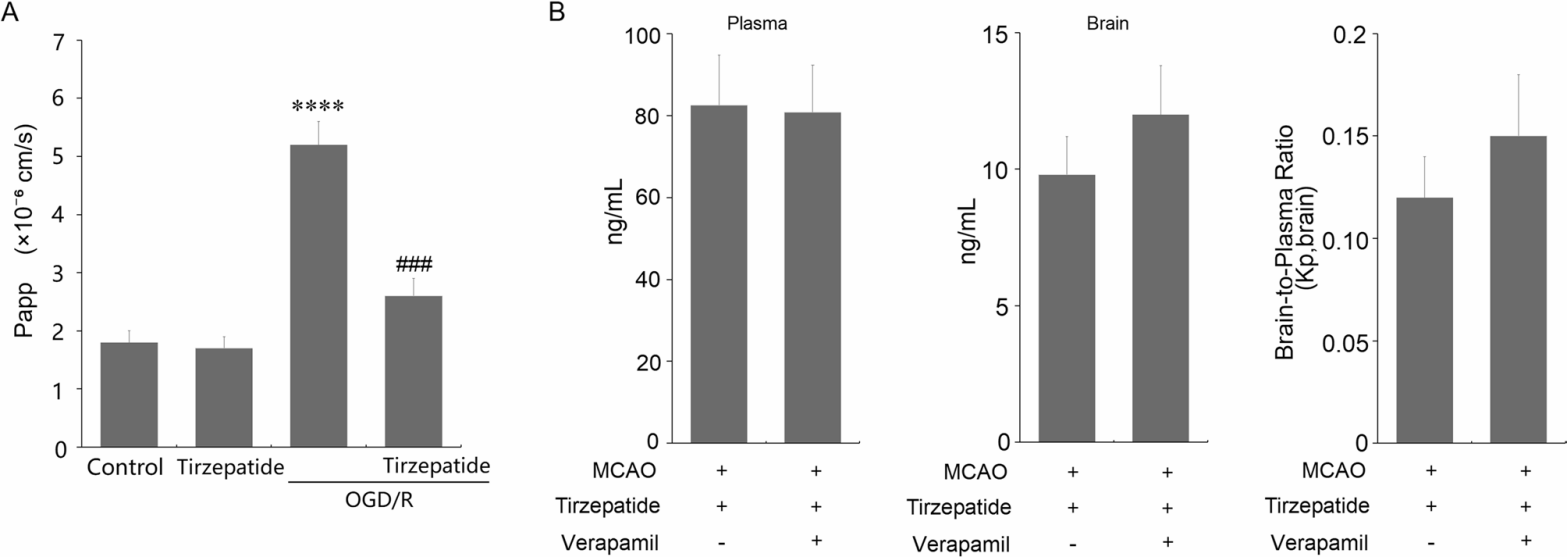
In Vitro and In Vivo BBB Permeability Assessments (**A**) Apparent permeability of FITC-dextran across HBMVEC monolayers under Control (0.1% DMSO), TZP (100 nM), OGD/R (2 h OGD, 24 h reperfusion), and OGD/R + TZP (100 nM) conditions (****P < 0.001 vs. Control, ^###^*P* < 0.005 vs. OGD/R, ANOVA with Tukey post hoc). (**B**, left) Plasma concentration of TZP in MCAO + TZP and MCAO + TZP + Verapamil groups (*P* > 0.05). (B, middle) Brain concentration in MCAO + TZP and MCAO + TZP + Verapamil groups (*P* > 0.05). (**B**, right) Brain-to-plasma ratio in MCAO + TZP and MCAO + TZP + Verapamil groups (*P* > 0.05)

To further evaluate TZP’s effects on BBB integrity, an in vitro Transwell assay measured FITC-dextran permeability in HBMVECs. OGD/R significantly increased permeability to 5.2 ± 0.4 × 10⁻⁶ cm/s compared to Control (1.8 ± 0.2 × 10⁻⁶ cm/s, *P* < 0.001, ANOVA with Tukey post hoc), reflecting stroke-induced endothelial barrier disruption. TZP treatment in OGD/R + TZP reduced permeability to 2.6 ± 0.3 × 10⁻⁶ cm/s (*P* < 0.005 vs. OGD/R), indicating robust protection of endothelial tight junctions (TJs). TZP alone (1.7 ± 0.2 × 10⁻⁶ cm/s) showed no effect on baseline permeability (*P* = 0.81 vs. Control; Fig. 10A). TEER post-assay remained > 150 Ω·cm² across groups, confirming monolayer integrity. These in vitro findings parallel the in vivo reduction of the ^14^C-sucrose influx in MCAO + TZP mice (Fig. 2A), suggesting consistent BBB protection. In vivo, the role of P-gp efflux in TZP’s BBB penetration was assessed (*n* = 6 mice per group). Verapamil (a P-gp inhibitor, 20 mg/kg) did not significantly alter plasma TZP concentrations (80 ± 11 ng/mL in MCAO + TZP + Verapamil vs. 82 ± 12 ng/mL in MCAO + TZP, *P >* 0.05; Fig. 10B, lft). Brain concentrations increased slightly with verapamil (12.0 ± 1.8 ng/g vs. 9.8 ± 1.4 ng/g, *P* > 0.05; Fig. 10B, middle). Similarly, the increase in Kp, brain from 0.12 ± 0.02 to 0.15 ± 0.03 (*P* > 0.05; Fig. 10B, right) was also not significant. In summary, both in vitro and in vivo experiments demonstrated that TZP effectively protects BBB integrity by reducing permeability and enhancing tight junctions, with minimal reliance on P-gp efflux for its CNS penetration.

## Discussion

ECs play a crucial role in the BBB, constituting the main part of the BBB and mediating the exchange of substances between brain capillaries and brain tissue (Yuan et al. 2023). These specialized brain ECs are distinct from those in peripheral tissues; their membranes are continuous and sealed, closed by additional intercellular junction structures of tight junction (TJ) proteins (Abdullahi et al. 2018). These unique structures confer on the BBB low transcellular transport, low paracellular flux, and high transendothelial electrical resistance, thereby effectively protecting the nervous system from harmful substances in the blood (Zhao et al. 2022). ECs of the BBB are not only transporters of substances and information but also sources of informational molecules, constantly performing active work in maintaining barrier function and regulating microenvironmental homeostasis through paracrine, autocrine, or endocrine mechanisms (Langen et al. 2019; Abbott et al. 2006).

After an IS, the excessive production of ROS in ECs and inflammatory responses lead to the activation of matrix metalloproteinases (MMPs), particularly MMP-9, which results in the degradation of TJ proteins. This degradation disrupts the BBB, leading to the infiltration of inflammatory cells, exacerbating oxidative stress and glial cell activation, and consequently causing neuronal death (Mathias et al. 2024). The present study demonstrates that TZP mitigates stroke-induced BBB disruption in MCAO mice, potentially through modulation of Claudin-1 and C/EBP-α pathways. Consistent with Wei et al. (Wei et al. 2023), MCAO mice showed elevated neurological scores and BBB permeability (Ki = 2.582 ± 0.156 µL/g/min vs. 1.00 ± 0.028 µL/g/min in Sham, *P* < 0.001), which TZP reduced to 1.447 ± 0.103 µL/g/min (*P* < 0.005 vs. MCAO). These findings, illustrated in Fig. 2A, confirm TZP’s protective effect on BBB integrity, as ^14^C-sucrose influx rates indicate significant restoration of barrier function in MCAO + TZP mice compared to MCAO alone. Congruent with data claimed by Wei et al. (Wei et al. 2023), increased neurological scores in MCAO mice were accompanied by enlarged BBB permeability, which was noticeably alleviated by TZP, implying a potential anti-stroke property of TZP via protecting BBB function. Aligned with Zeng et al. (Zeng et al. 2020), enhanced endothelial permeability was triggered in HBMVECs under OGD/R stimulation, which was notably rescued by TZP, hinting that TZP might protect the BBB by restoring endothelial permeability and function. This is further supported by an in vitro Transwell assay, where TZP reduced OGD/R-induced permeability in HBMVECs (*P* < 0.005, Fig. 10A), suggesting a direct protective effect on endothelial tight junctions.

Claudin-1 is a transmembrane protein and one of the main components along with other members of the claudin family, plays a role in TJs, which is crucial for maintaining BBB function (Bony et al. 2021). TJs are intercellular adhesion complexes that act as gatekeepers of the paracellular space, with the claudin protein family performing the primary gatekeeping functions (Marsch et al. 2024). Claudin-1 is engaged in regulating TER and paracellular permeability, thereby affecting the barrier function of TJs (Wang et al. 2020). Corresponding to results reported by Chen (Chen et al. 2021), BBB dysfunction in MCAO animals and OGD/R-evoked endothelial permeability enlargement were accompanied by downregulated Claudin-1 levels, which were remarkably enhanced by TZP, hinting that TZP might rescue BBB function by repairing TJ structure via upregulating Claudin-1.

C/EBP-α is a key transcription factor that regulates the differentiation of various cell types and interacts with cyclin proteins (Nerlov 2007). As a transcription factor, C/EBP-α can enter the cell nucleus and further directly or indirectly regulate multiple genes that are key factors in cell functions (Song et al. 2015). The role of C/EBP-α is particularly important in the regulation of TJ protein expressions. JAM-A positively regulates C/EBP-α through the exchange protein directly activated by the cAMP (EPAC) pathway, thereby upregulating TJ protein Claudin-5 and reducing the permeability of endothelial cells (Kakogiannos et al. 2020). Herein, both in MCAO mice and OGD/R-managed ECs, decreased Claudin-1 expressions were accompanied by downregulated C/EBP-α, expressions of which were remarkably reversed by TZP, suggesting that TZP might maintain TJ function and Claudin-1 expressions via activating the C/EBP-α signaling. Furthermore, influences of TZP on BBB permeability and Claudin-1 expressions in MCAO mice, as well as on endothelial permeability and Claudin-1 expressions in OGD/R-managed ECs, were noticeably abolished by silencing C/EBP-α, confirming that TZP restored Claudin-1-mediated TJ and BBB function via activating the C/EBP-α signaling.

To benchmark TZP’s BBB permeability profile, TZP’s brain-to-plasma ratio (Kp, brain) was measured as 0.12 ± 0.02 in healthy mice (TZP group) and 0.14 ± 0.02 in stroke mice (MCAO + TZP), both significantly higher than liraglutide’s 0.03 ± 0.01 (*P* < 0.01) (Rhea et al. 2024), as shown in Fig. 2B. This fourfold increase suggests TZP’s dual GIP/GLP-1 agonism and fatty acid modification enhance CNS penetration compared to single GLP-1 agonists like liraglutide, which is restricted to circumventricular organs. The slight Kp, brain increase in MCAO + TZP (*P* > 0.05 vs. TZP) may reflect stroke-induced BBB leakiness, consistent with elevated ^14^C-sucrose uptake in MCAO (Fig. 2A). Additionally, P-glycoprotein (P-gp) inhibition with verapamil slightly increased TZP’s Kp, brain to 0.15 ± 0.03 (*P* > 0.05 vs. MCAO + TZP, Fig. 10B), indicating minimal efflux limitation, further distinguishing TZP from liraglutide, which is more P-gp restricted (Rhea et al. 2024). These results, illustrated in Figs. 2B and 10B, highlight TZP’s superior CNS access, likely contributing to its efficacy in stabilizing BBB function.

TZP demonstrates robust protective effects on the BBB in ischemic stroke models. In vitro, TZP reduced OGD/R-induced endothelial permeability in HBMVECs (*P* < 0.005, Fig. 10A), indicating direct stabilization of endothelial tight junctions. In vivo, P-glycoprotein (P-gp) inhibition with verapamil slightly increased TZP’s Kp, brain to 0.15 ± 0.03 (*P* = 0.08 vs. MCAO + TZP, Fig. 10B), suggesting minimal efflux limitation compared to liraglutide, which exhibits restricted CNS access consistent with P-gp efflux (Secher et al. 2014). These findings complement TZP’s ability to reduce stroke-induced BBB permeability (Ki: 1.447 ± 0.103 µL/g/min in MCAO + TZP vs. 2.582 ± 0.156 µL/g/min in MCAO, *P* < 0.005, Fig. 2A) and enhance CNS penetration (Kp, brain: 0.12–0.14, Fig. 2B) through C/EBP-α-mediated restoration of Claudin-1.

Our study utilized [¹⁴C]-sucrose influx rates and LC-MS/MS for Kp, brain quantification, however certain limitations should be noted. The [¹⁴C]-sucrose assay primarily reflects paracellular leakage rather than active transport mechanisms. Potential matrix effects during brain homogenate analysis or incomplete drug extraction could also influence accuracy. Future studies incorporating multiple time points and advanced imaging techniques (e.g., PET) would strengthen these findings.

Although enhanced BBB penetration may amplify TZP’s neuroprotective efficacy, it raises concerns about CNS-related adverse effects. Clinical trials report nausea as a common side effect of GLP-1 agonists, which could be exacerbated by increased brain exposure (Jastreboff et al. 2022). While our MCAO model showed no overt behavioral toxicity, long-term studies are warranted to evaluate TZP’s safety profile, particularly in non-stroke contexts where BBB integrity is intact. Furthermore, the contribution of efflux transporters (e.g., BCRP) to TZP’s CNS distribution remains unexplored and merits investigation. Although we hypothesize receptor-mediated transport for TZP’s BBB crossing, based on C/EBP-α activation, direct evidence to support this mechanism remains to be obtained. Future studies should incorporate direct blood flow measurements to further validate the ischemic conditions. Future studies using radiolabeled TZP and transporter inhibitors (e.g., P-glycoprotein inhibitors) are needed to confirm this mechanism.

## Conclusions

In summary, TZP’s observed enhancement in BBB permeability and its capacity to reduce BBB leakage suggests potential promise as a therapeutic candidate for stroke, warranting further exploration to fully establish its clinical applicability. Its ability to restore Claudin-1-mediated TJ and BBB function through the activation of C/EBP-α signaling offers a potential approach to protect against stroke-induced neuronal damage. Further investigation into the mechanisms of TZP action and its potential clinical applications could lead to the development of more effective treatments for stroke, ultimately improving patient outcomes.

## Supplementary information

Supplementary Material 1.

Supplementary Material 2.

Supplementary Material 3.

Supplementary Material 4.

Supplementary Material 5.

Supplementary Material 6.

Supplementary Material 7.

Supplementary Material 8.

## Data Availability

The data used during the current study are available from the corresponding author on reasonable request.
