# Supplementary figures and images for: Tirzepatide mitigates Stroke-Induced Blood-Brain barrier disruption by modulating Claudin-1 and C/EBP-α pathways
Source: Mol Med. 2025 Jul 23;31:263. doi: 10.1186/s10020-025-01312-4 (PMC12285096; doi:10.1186/s10020-025-01312-4)

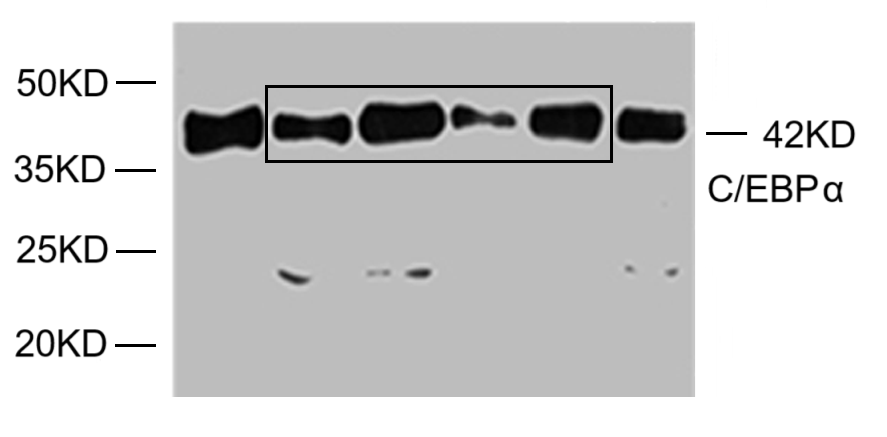

Supplement: Supplementary file 1 — Supplementary Material 1. [file 10020_2025_1312_MOESM1_ESM.tif]

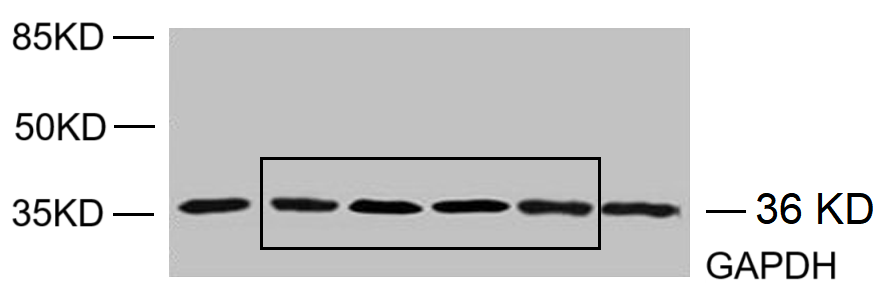

Supplement: Supplementary file 2 — Supplementary Material 2. [file 10020_2025_1312_MOESM2_ESM.tif]

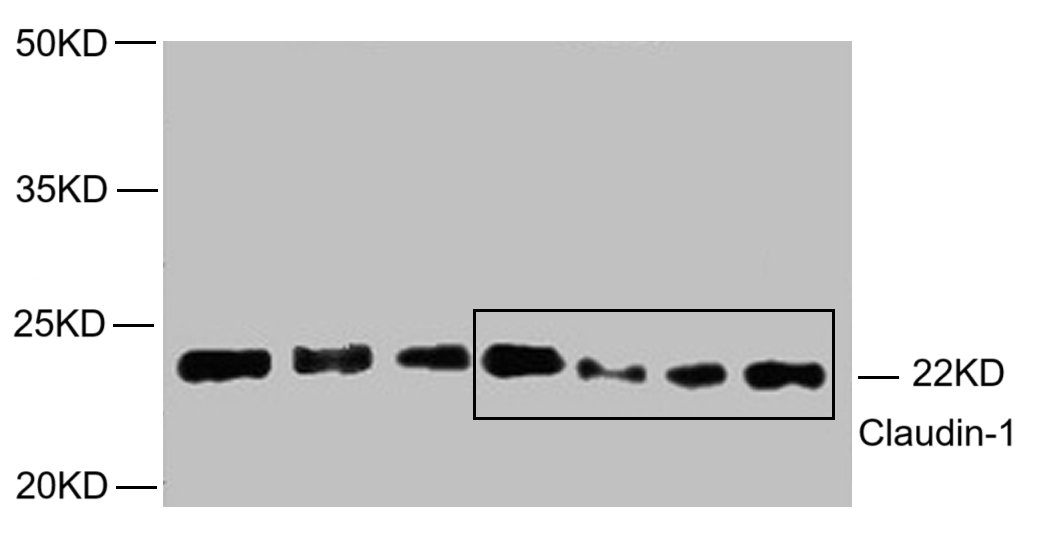

Supplement: Supplementary file 3 — Supplementary Material 3. [file 10020_2025_1312_MOESM3_ESM.tif]

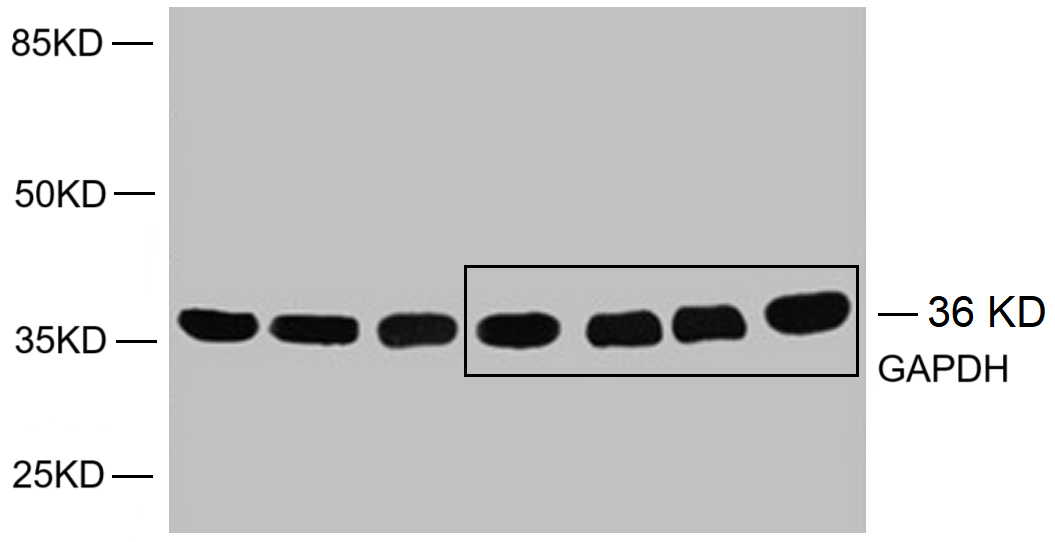

Supplement: Supplementary file 4 — Supplementary Material 4. [file 10020_2025_1312_MOESM4_ESM.tif]

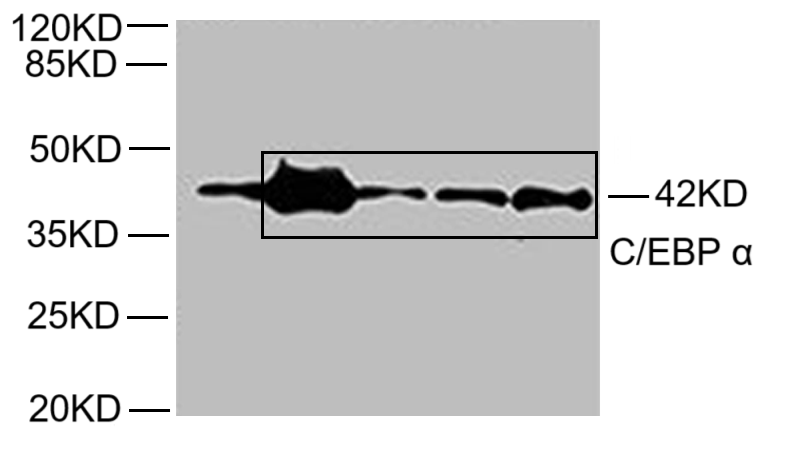

Supplement: Supplementary file 5 — Supplementary Material 5. [file 10020_2025_1312_MOESM5_ESM.tif]

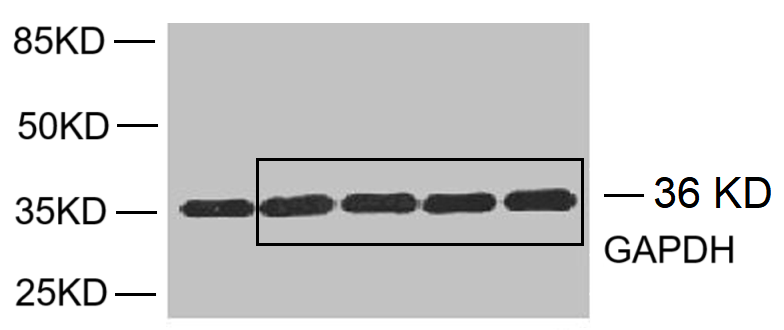

Supplement: Supplementary file 6 — Supplementary Material 6. [file 10020_2025_1312_MOESM6_ESM.tif]

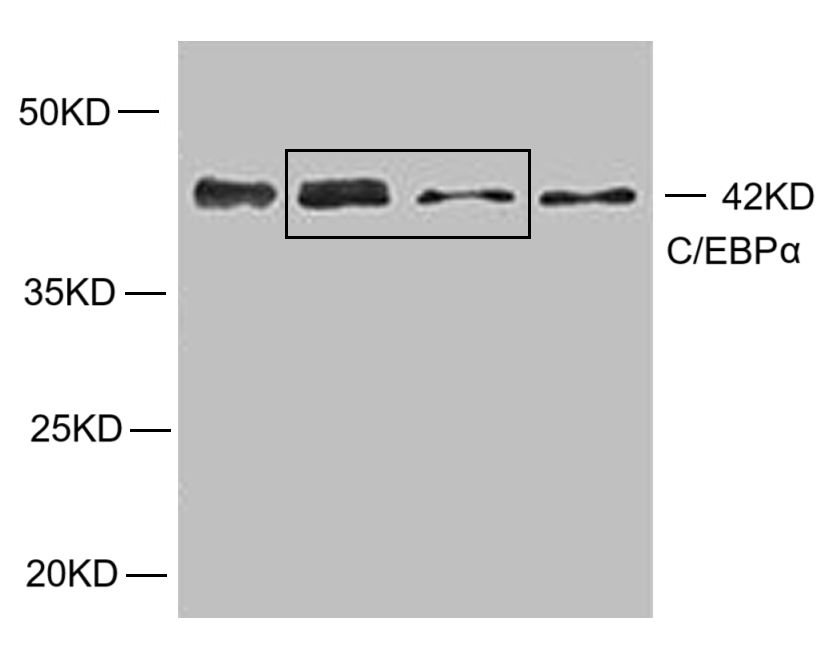

Supplement: Supplementary file 7 — Supplementary Material 7. [file 10020_2025_1312_MOESM7_ESM.tif]

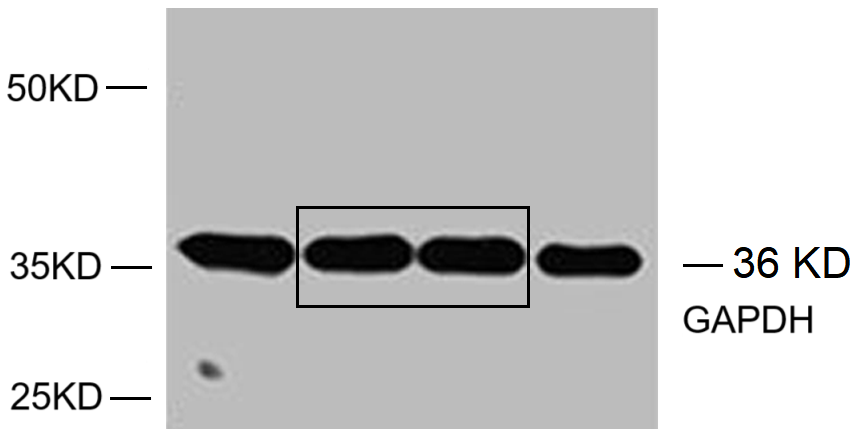

Supplement: Supplementary file 8 — Supplementary Material 8. [file 10020_2025_1312_MOESM8_ESM.tif]
